# Revealing the dynamic landscape of drug-drug interactions through network analysis

**DOI:** 10.3389/fphar.2023.1211491

**Published:** 2023-10-03

**Authors:** Eugene Jeong, Bradley Malin, Scott D. Nelson, Yu Su, Lang Li, You Chen

**Affiliations:** ^1^ Department of Biomedical Informatics, School of Medicine, Vanderbilt University Medical Center, Nashville, TN, United States; ^2^ Department of Biostatistics, School of Medicine, Vanderbilt University Medical Center, Nashville, TN, United States; ^3^ Department of Computer Science, School of Engineering, Vanderbilt University, Nashville, TN, United States; ^4^ Department of Computer Science and Engineering, College of Engineering, The Ohio State University, Columbus, OH, United States; ^5^ Department of Biomedical Informatics, College of Medicine, The Ohio State University, Columbus, OH, United States

**Keywords:** pharmacokinetic drug-drug interaction, pharmacodynamic drug-drug interaction, network analysis, natural language Processing, research trend

## Abstract

**Introduction:** The landscape of drug-drug interactions (DDIs) has evolved significantly over the past 60 years, necessitating a retrospective analysis to identify research trends and under-explored areas. While methodologies like bibliometric analysis provide valuable quantitative perspectives on DDI research, they have not successfully delineated the complex interrelations between drugs. Understanding these intricate relationships is essential for deciphering the evolving architecture and progressive transformation of DDI research structures over time. We utilize network analysis to unearth the multifaceted relationships between drugs, offering a richer, more nuanced comprehension of shifts in research focus within the DDI landscape.

**Methods:** This groundbreaking investigation employs natural language processing, techniques, specifically Named Entity Recognition (NER) via ScispaCy, and the information extraction model, SciFive, to extract pharmacokinetic (PK) and pharmacodynamic (PD) DDI evidence from PubMed articles spanning January 1962 to July 2023. It reveals key trends and patterns through an innovative network analysis approach. Static network analysis is deployed to discern structural patterns in DDI research, while evolving network analysis is employed to monitor changes in the DDI research trend structures over time.

**Results:** Our compelling results shed light on the scale-free characteristics of pharmacokinetic, pharmacodynamic, and their combined networks, exhibiting power law exponent values of 2.5, 2.82, and 2.46, respectively. In these networks, a select few drugs serve as central hubs, engaging in extensive interactions with a multitude of other drugs. Interestingly, the networks conform to a densification power law, illustrating that the number of DDIs grows exponentially as new drugs are added to the DDI network. Notably, we discovered that drugs connected in PK and PD networks predominantly belong to the same categories defined by the Anatomical Therapeutic Chemical (ATC) classification system, with fewer interactions observed between drugs from different categories.

**Discussion:** The finding suggests that PK and PD DDIs between drugs from different ATC categories have not been studied as extensively as those between drugs within the same categories. By unearthing these hidden patterns, our study paves the way for a deeper understanding of the DDI landscape, providing valuable information for future DDI research, clinical practice, and drug development focus areas.

## 1 Introduction

Drug-drug interactions (DDIs) occur when the effect of one drug is altered by the presence of another drug (van Mil, 2016). DDIs can be broadly classified into two types: 1) pharmacokinetic (PK), which occurs when one drug modifies the disposition (i.e., absorption, distribution, metabolism, and/or excretion) of another drug (Nebert and Russell, 2002; Nigam, 2015), and 2) pharmacodynamic (PD), which occur when the pharmacological effects (on cells, organs, and systems) of one drug are altered or additive by the presence of another (Niu et al., 2019). These interactions can generate a wide range of outcomes, often causing adverse effects and deteriorating patients’ health. Consequently, DDIs have been the subject of numerous studies over the past several decades, with progress in high-throughput screening methods, the rapid growth of biomedical databases, and an increase in clinical studies contributing to the discovery of novel DDIs and insights into their underlying PK and PD mechanisms (Becker et al., 2007).

The vast amount of data generated by the numerous studies on DDIs has made it challenging for researchers to analyze research trends and evolutions, which makes it difficult to gain a comprehensive understanding of the overall landscape of DDIs, identify under-explored areas, discern research trends, and pinpoint areas of focused interest. To address this issue, some studies have used bibliometric analysis (Wang et al., 2022; Sun et al., 2022; Pirri et al., 2020; KURUTKAN, 2023), a quantitative method that evaluates and analyzes various aspects of scientific publications. Bibliometric indicators such as the number of publications, citations, and authors can provide a valuable quantitative overview of DDI research. However, this approach has limitations in its ability to capture the complex relationships between drugs and the evolving nature of DDI research, despite its numerical precision and ease of use.

To thoroughly examine the DDI research landscape, we constructed DDI networks based on evidence extracted from PubMed article abstracts by natural language processing (NLP) models and analyzed them using network analysis (NA). NLP models facilitate the automation of information extraction from extensive unstructured text data, enabling researchers to analyze large datasets more quickly and efficiently (Boyce et al., 2012). Network analysis, on the other hand, serves as a powerful model for analyzing complex interactions between drugs, providing a more comprehensive picture of the structure and allowing researchers to represent and explore complex data in a more intuitive and accessible way (Jeong et al., 2017; Chen et al., 2020; Yan et al., 2021).

By utilizing DDI networks, we can gain a complete understanding of the DDI research landscape and the chronological development of the field. This approach provides a comprehensive view of the dynamic landscape of drug-drug interactions and allows for the identification of shifts in the DDI landscape. Our integration of NLP and NA allows researchers to identify areas of focused interest and under-explored areas, recognize emerging areas of concern or novel research trajectories, and spot gaps in the field that may harbor potential yet under-studied drug interactions. Ultimately, this approach may inform decision-making in drug development, clinical practice, and DDI research prioritization.

## 2 Materials and methods

### 2.1 Retrieving DDI evidence from PubMed abstracts

**FIGURE 1:**
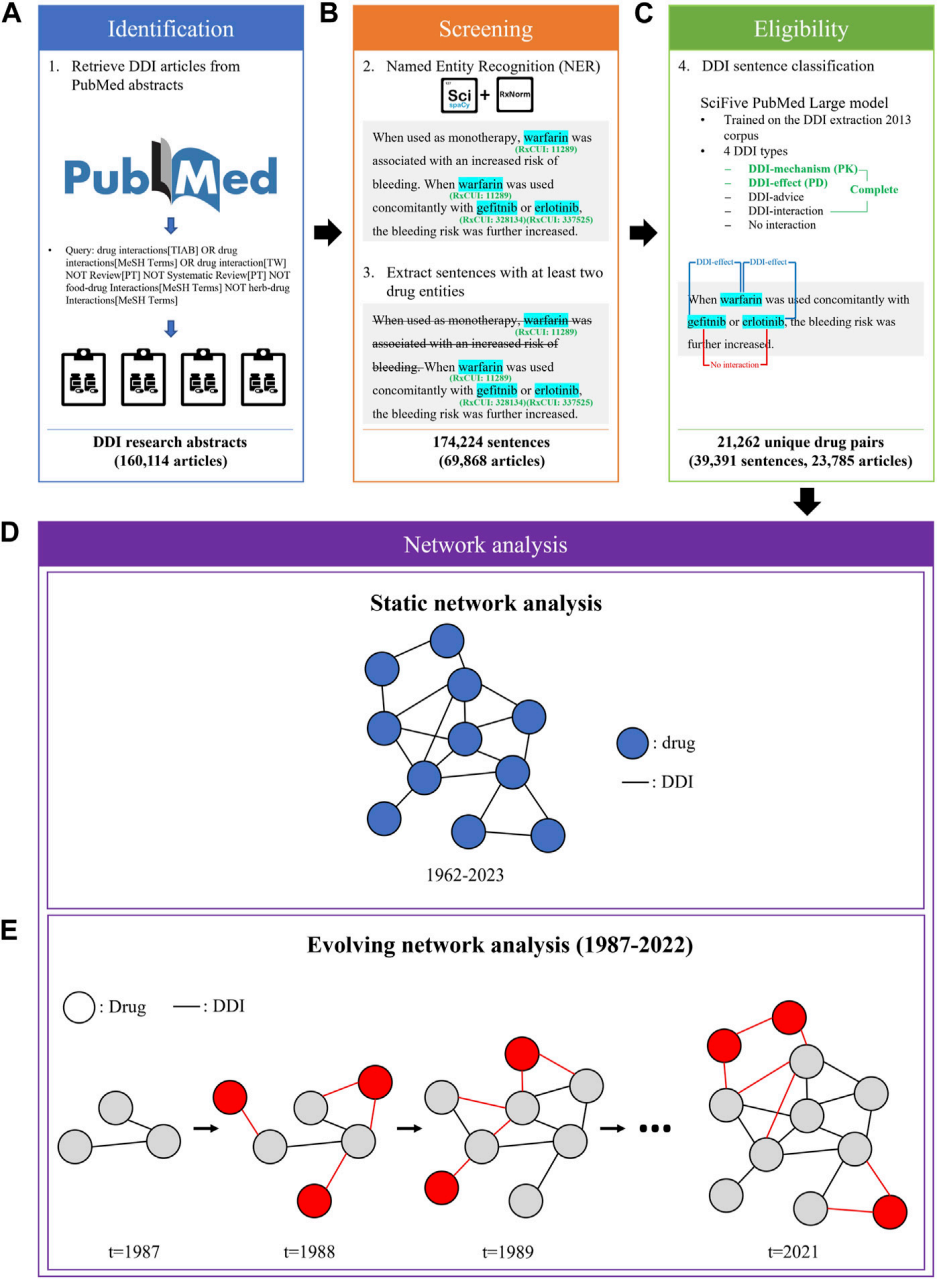
The process of DDI evidence extraction and dynamic network analysis**. (A)** A custom query was employed to retrieve articles related to DDIs from PubMed API. **(B)** Sentences with at least two drug entities from abstracts were extracted using the ScispaCy NER model. **(C)** The SciFive DDI RE model was applied to extract DDIs from DDI sentences. **(D)** A static network was constructed based on the entire extracted DDI sentences from 1962 to 2023. **(E)** An evolving network analysis was conducted to examine the 36-year trend in DDI research.

**FIGURE 2:**
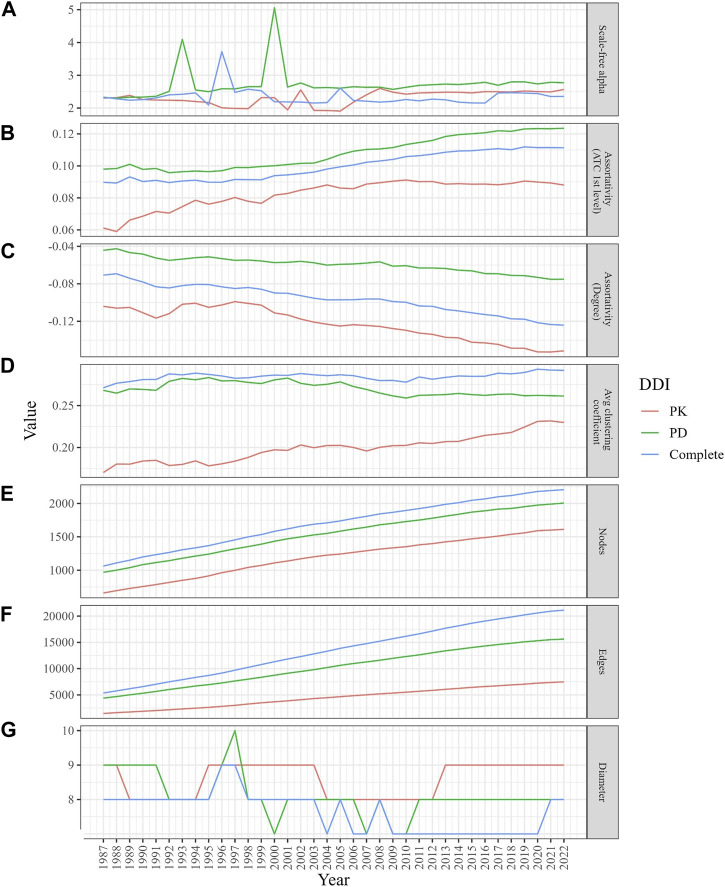
Changes in the structural properties of the evolving DDI networks (PK, PD, and complete) from 1987 to 2022. **(A)** The graph demonstrates a stable scale-free alpha (between 2 and 3) for the PK DDI network, while the PD and complete DDI networks show fluctuations until 2001. **(B)** The graph displays a higher likelihood of DDIs among drugs in the same ATC category. **(C)** The graph indicates a decrease in degree assortativity. **(D)** The graph indicates an increase in local clustering coefficients, especially in the PD network. **(E**,**F)** The graphs illustrate how the size of the network grows over time. **(G)** Despite network growth, the diameters remained stable.

**FIGURE 3:**
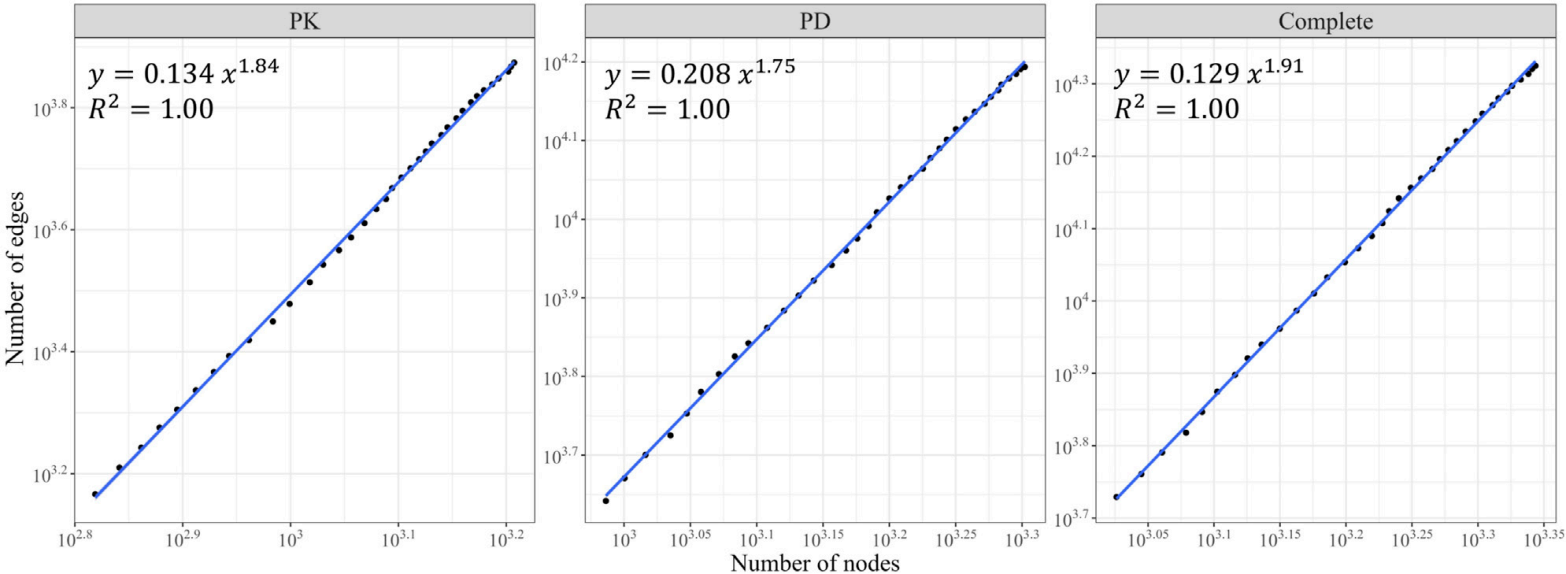
The Densification Power Law. The number of edges is plotted against the number of nodes for the PK, PD, and complete DDI networks on a log-log scale. All three networks exhibited a densification power law with a rapid rate of interaction growth (high densification exponents).

We applied a three-step procedure to collect evidence on DDIs from PubMed abstracts published between January 1962 and July 2023: 1) identification of candidate articles about DDIs using a PubMed query (Figure 1A), 2) screening of the articles containing sentences with drug entities using a named entity recognition (NER) model (Figure 1B), and 3) determination of eligible sentences about DDIs using a relation extraction (RE) model (Figure 1C).

#### 2.1.1 PubMed query

We designed a query in accordance with Duda et al. (2005) to retrieve a set of DDI articles with high sensitivity, the broadest search to include all DDI-relevant articles: “drug interactions” [TIAB] OR “drug interactions” [MeSH Terms] OR “drug interaction” [TW] NOT food-drug interactions [MeSH Terms] NOT herb-drug interactions [MeSH Terms] NOT Review [PT] NOT Systematic Review [PT]. This query was chosen to ensure that no pertinent documents were missed.

#### 2.1.2 NER model

In order to search for evidence of DDIs in retrieved articles, it is necessary to first identify drug entities within sentences. To accomplish this, a NER model, a type of NLP model, that is, used to identify and extract entities, is required for the efficient and accurate detection of drug entities.

The SpaCy Python library is an open-source library designed to support a variety of tasks such as POS Tagging, NER, and Dependency Parsing (Honnibal and Montani, 2017). ScispaCy (Neumann et al., 2019) is an extension of spaCy developed for biomedical, scientific, or clinical text. It has become the *de facto* standard for practical NLP due to its speed, reliability, and near-state-of-the-art performance (SOTA). Entity linker is a SciSpacy feature that maps entities mentioned in the text to standard, canonical identifiers in a knowledge base or database. These databases for biomedical texts could include UMLS (Unified Medical Language System), RxNorm, and others. The linker conducts a string overlap-based search (char-3grams) on named entities, comparing them with the concepts in a knowledge base via an approximate nearest neighbors search. We implemented RxNorm entity linker in the ScispaCy, which contains ∼100 k concepts focused on normalized names for clinical drugs. The ScispaCy-large model was used to perform NER, sentence tokenization, and entity-linking features for every sentence from abstracts. Given that the ScispaCy only provides CUI for RxNorm entities, we used the MRCONSO.RRF file from UMLS Metathesaurus (Bodenreider, 2004) to map CUI to RxCUI. To minimize the chance of duplicating clinically similar RxNorm concepts, we further linked RxNorm concepts to RxNorm ingredients using the RXNREL.RRF file from UMLS Metathesaurus. Finally, we extracted sentences containing at least two drug entities for further analysis of potential DDIs.

#### 2.1.3 RE model

To identify eligible evidence of DDIs from sentences containing at least two drug entities, a RE model, a type of NLP model, that is, used to identify and extract the relationships between entities in text, was utilized to find the DDI relationship within the sentences. The SciFive PubMed Large model is a domain-specific text-to-text transfer transformer (T5) model (Raffel et al., 2020) that is, pre-trained on PubMed abstracts using 1.2 million steps to optimize the pre-trained weights from the T5 model in the context of biomedical literature. The DDI extraction 2013 corpus is comprised of 792 texts taken from the DrugBank database and 233 Medline papers; it has been created for the SemEval 2013 DDIExtraction challenge, whose primary objective is to provide a common framework for the evaluation of information extraction techniques applied to the recognition of pharmacological substances and the detection of DDIs from biomedical texts, and has been used as the gold standard for evaluating DDI extraction task performance (Segura-Bedmar et al., 2013). Two expert pharmacists with extensive experience in pharmacovigilance annotated drug-drug interactions, covering both pharmacokinetic and pharmacodynamic interactions. The five classifications consist of four distinct types of interactions and one type of non-interaction in the corpus, as follows: 1) No interaction: a sentence does not represent an interaction between two drugs, 2) DDI-mechanism: a sentence describes a pharmacokinetic mechanism, 3) DDI-effect: a sentence describes the effect of the DDI or pharmacodynamic mechanism, 4) DDI-advice: a sentence describes a recommendation or advice regarding a drug interaction, and 5) DDI-int: a sentence describes a drug interaction without providing any other information. The SciFive PMC Large model achieved a level of performance that was similar to SOTA on DDI relation extraction using the DDI extraction 2013 corpus (precision: 83.88, recall: 83.45, and F1 score: 83.63). We applied the pre-trained weights of the SciFive PMC Large model distributed by the authors (Phan et al., 2021), and further fine-tuned the model parameters using the DDI extraction 2013 corpus to determine the reliability of each candidate DDI evidence. If a candidate DDI sentence contained more than two RxNorm ingredients, all possible drug-drug combinations were investigated, implying that a single sentence could contain both a drug pair that does not interact and a drug pair that does interact.

To validate the performance of the SciFive model, we randomly selected extracted DDI evidence and manually annotated them with the help of two reviewers (one with an M.S. in biomedical informatics and one with a Ph.D. in computer science). Both reviewers had 3 years of experience in drug-interaction research. The level of agreement between the two reviewers was measured using Cohen’s Kappa.

#### 2.1.4 Mapping RxNorm ingredients to ATC first levels

RxNorm ingredients were mapped to first-level Anatomical Therapeutic Chemical (ATC) classes using the RxNorm API (https://mor.nlm.nih.gov/RxNav/) for drug classification purposes. The ATC first level contains 14 major anatomical or pharmacological groups. If a RxNorm ingredient had multiple ATC first levels, then all ATCs were counted separately. If a RxNorm ingredient was unmapped to any ATC first levels, then it was assigned to a “No ATC” group.

### 2.2 Network construction

#### 2.2.1 Static networks

Based on all extracted DDI sentences from 1962 to 2023, we constructed three static networks: 1) one for PKs, 2) one for PDs, and 3) one for the complete set of DDIs, including PK and PD, as well as those classified as DDI-advice or DDI-int (Figure 1D). In such networks, each node represents a drug and each edge exists between two nodes if there was at least one sentence from the literature with evidence of a DDI between the two drugs.

#### 2.2.2 Evolving networks

To model the dynamic changes in the DDI networks, we created evolving networks of drugs based on DDIs extracted from each year (Figure 1E). The network T_*i+1*_ is an augmentation of the prior network T_*i*_, where *i* represents the year. For example, the network of 1988 represented the addition of new drugs and DDIs published in 1988 to the network of 1987. Similarly, the network of 1989 expanded upon the 1988 network, and this pattern continued in subsequent years. Due to a lack of sufficient data to create yearly networks for years prior to 1987, we chose 1987 as the earliest investigated year for the evolving network analysis. In addition, we have excluded 2023 data from the evolving network analysis due to lacking data for the entire year.

### 2.3 Network-level properties

In order to provide a more comprehensive understanding of the structure of DDI research, we measured various network structural properties in this study. These properties included the number of nodes and edges, assortativity based on degree and ATC first level categories, average local clustering coefficient, power law exponent γ, network diameter, and the densification power law (DPL). The number of nodes and edges was specifically measured to gain insights into the size of the networks. The degree assortativity is the tendency for nodes of high degree (resp. low degree) in a graph to be connected to high degree nodes (resp. to low degree ones), while ATC-group assortativity is the tendency for nodes to be connected to drugs in the same ATC categories. The average local clustering coefficient measures how close its neighbors are to form a clique. If the neighborhood is fully connected, the clustering coefficient is 1, whereas a value close to 0 indicates that the neighborhood has few connections. The diameter of a network is defined as the smallest distance between the two furthest nodes in the network. This distance is determined by computing the shortest path length between every node and all other nodes and selecting the longest path length as the network’s diameter. A smaller network diameter suggests that the drugs in the network are more closely related and may have a higher potential for interactions, while a larger diameter may indicate that the drugs are more diverse and less likely to interact. To determine whether the number of edges grows faster than the number of nodes in the networks, we measured the DPL. The DPL is a concept from the temporal graph evolution (Leskovec et al., 2007) domain. This law indicates that the number of edges should grow according to a power law over the number of nodes over time:

$${e\left(t\right)\propto n\left(t\right)}^{a}$$ (1)

where $e\left(t\right)$ and *n*(*t*) denote the number of edges and nodes, respectively, of the graph at time *t*, and *a* is an exponent (*a* = 1 represents a constant average degree throughout time, whereas *a* = 2 represents to an extremely dense graph in which each node has edges to a constant fraction of all nodes on average.) Numerous studies have shown that many real-world evolving networks exhibit a densification power law property (Leskovec et al., 2005; Leskovec et al., 2007; Qu et al., 2014; Qu et al., 2015). Network analysis was conducted using the *igraph* package in R (Csardi and Nepusz, 2006).

### 2.4 ATC categories-level properties

Our analysis focuses on DDI networks at the level of ATC classification groups. We aim to determine whether the observed DDI interactions occur within the same therapeutic class or across multiple classes. This approach allows us to investigate the potential for interactions between drugs with similar or different mechanisms of action and may provide insights into the overall safety and efficacy of drug combinations within specific therapeutic categories.

#### 2.4.1 The Krackhardt E/I ratio

The Krackhardt E/I Ratio (Krackhardt and Stern, 1988), also known as the E-I index, is a measure of homophily that quantifies the extent to which one node is linked to similar or dissimilar nodes. The E-I index is computed as:

$$E-I\;index=\frac{EL-IL}{EL+IL}$$ (2)

where EL and IL denote the number of external links and internal links, respectively. The E-I index ranges from −1 to 1, and if it is positive, it indicates that there are more external links than internal links (heterophily). If the value is close to 0, it indicates that links are distributed equally; and if it is negative, it indicates that there are more internal links than external links (homophily).

#### 2.4.2 Fisher’s exact test for ATC-ATC pairs

To determine the most interconnected pairs of ATC categories (those with a higher chance of having DDIs between drugs from the two categories compared to other categories), all possible ATC category-ATC category combinations were extracted from the network and generated in the 2-by-2 contingency table (Table 1). Numbers are assigned to one of the contingency table cells based on the number of interactions between ATC categories. For example, *a* denotes the number of interactions between the #1 category and #2 category, and b denotes the number of interactions that the #1 category has with ATC categories other than the #2 category. A Fisher’s exact test with Bonferroni correction was relied upon to determine significance. The ATC-ATC pairs with *p*-values less than 0.05 after Bonferroni correction and odds ratios greater than 1 were considered statistically significant.

**TABLE 1:** Two-by-two contingency table for evaluating ATC 1-ATC 2 pairs.

	ATC category #2	No ATC category #2
ATC category #1	a	b
No	c	d
ATC category #1		

### 2.5 Drug-level properties

While ATC-group-level analyses examine classes of drugs, drug-level analyses focus on individual drugs. This approach provides a more detailed understanding of specific drug interactions and is essential for identifying key drugs in the DDI network. By examining the interactions of individual drugs, we can gain insights into the mechanisms of action that underlie drug interactions and identify drugs that are more likely to be involved in multiple interactions.

#### 2.5.1 Centrality measures

In network analysis, several types of centrality measures can be used to understand the relative importance of drugs within the DDI network. In this study, we concentrated on three centrality measures: degree centrality, betweenness centrality, and eigenvector centrality. The degree centrality is a simple centrality measure that counts how many neighbors a drug has, finding drugs that are likely to be the center and can quickly connect with the wider network. The betweenness centrality measures the number of times a drug lies on the shortest path between other drugs. This measure shows which drugs are bridges between drugs in a network, showing drugs that influence the flow in the DDI network. Eigenvector centrality measures a drug’s influence based on the number of links it has to other drugs in the network. A high eigenvector score means that a drug is connected to many drugs that themselves have high scores.

#### 2.5.2 Emerging and declining drugs in the DDI research field

To identify drugs that have recently emerged in DDI research, we calculated the degree, betweenness, and eigenvector centrality growth rates for each drug in the yearly networks over the past 5 years (2018–2022). Drugs with a rapid growth rate are likely to be part of a new trend, attracting increased attention in recent years. The growth rate (slope) was estimated using linear regression. To identify the drugs that are receiving less attention in DDI research, we analyzed the lowest increase or highest decrease rate in centrality measures.

## 3 Results

### 3.1 The DDI sentences extracted from PubMed

We retrieved 160,114 candidate articles from the PubMed API through a search query designed for high sensitivity. Next, we applied ScispaCy for NER and extracted 174,224 sentences (69,868 articles) that contained at least two drug entities from the abstracts of the DDI articles. Finally, we used the SciFive model to extract 2,212 unique drugs and 21,262 unique DDIs (PK: 7,579, PD: 15,676) from 174,224 sentences. Among the 21,262 unique DDIs, 2,445 exhibited both PK and PD DDIs (Supplementary Figures S1, S2). To validate the performance of the SciFive model, we randomly selected 1,296 DDIs (36 for each year from 1987 to 2022) from the 21,233 DDIs. The level of agreement between the two reviewers was found to be extremely high with κ = 0.95; *p* < 0.001. The SciFive model achieved F1 scores of 0.892. All DDI sentences are provided in Supplementary Table S1.

### 3.2 Static network analysis

#### 3.2.1 An analysis of static DDI networks reveals scale-free structure, ATC category-based assortativity, and degree-based dissortativity

Table 2 shows the structural properties of the static DDI networks. All PK, PD, and complete DDI networks were scale-free (2 < γ < 3), with power law exponents (γ) of 2.56, 2.77, and 2.36. This indicates that a small number of drugs had many connections to other drugs, while most drugs had relatively few DDIs. Additionally, in the three networks, the ATC category-based assortativities were positive, suggesting that drugs from the same ATC category were more commonly investigated for DDIs than those from different ATC categories. Moreover, all three networks showed negative degree assortativity, meaning that few drugs were frequently confirmed to have DDIs with a large number of other drugs, each of which was rarely investigated to have a large number of DDIs.

**TABLE 2:** Structural network properties of the static PK, PD, and complete DDI networks.

Structural network property	PK	PD	Complete
Nodes	1,620	2,011	2,212
Edges	7,579	15,676	21,262
Assortativity (Degree)	−0.151	−0.0754	−0.124
Assortativity (ATC 1st level)	0.087	0.123	0.111
Power law exponent	2.5	2.82	2.36
Avg clustering coefficient	0.23	0.26	0.290
Diameter	9	8	8

#### 3.2.2 The average clustering coefficients reveal a prevalence of real DDIs among neighbors in static networks

The average clustering coefficients for the PK, PD, and complete networks are 0.23, 0.26, and 0.29, respectively. These are significantly higher than the clustering coefficients [0.005 (0.003–0.008), 0.007 (0.004–0.009), and 0.008 (0.006–0.011)] of random networks generated by Erdős-Rényi algorithms with the same number of nodes and edges. We performed 100 random network simulations.

The larger clustering coefficients of the PK, PD, and complete DDI networks suggest that about 30% of the potential connections among a drug’s neighbors in the network are actual DDIs. This means that, when examining the neighbors of a drug in the network, there is at least a 30% chance of finding a real DDI between them.

#### 3.2.3 Network diameter: comparing DDI static networks to the six degrees of separation

The diameter of all three networks, ranging from 8 to 9 (Table 2), slightly exceeds the well-known six degrees of separation observed in our world (Kleinfeld, 2002). The six degrees of separation theory is a concept that suggests any two people on Earth are, on average, separated by no more than six social connections, indicating that networks are both extensive and interconnected. As more drugs and their DDIs are investigated and added to the network, there may be a possibility of reducing the diameter from its current range to 6.

#### 3.2.4 ATC drug category E-I homophily index indicates a higher likelihood of DDIs within the same ATC drug category in static networks

The E-I homophily index values, which measure the degree to which a drug forms DDIs with others in the same category, were smaller than 1 (except for the V in the PD subnetwork) (Table 3). This suggests a tendency for drugs to establish connections with those belonging to the same group (homogeneous interactions).

**TABLE 3:** Network properties at the level of ATC category in the static PK, PD, and complete DDI networks.

		PK DDI network				PD DDI network				Complete DDI network			
ATC 1st level code	Anatomical or pharmacological groups	Node	Edge	E-I index	Sig. pair^a^ ^(OR)^	Node	Edge	E-I index	Sig. Pair ^(OR)^	Node	Edge	E-I index	Sig. Pair ^(OR)^
A	Alimentary tract and metabolism	1,515	2,456	0.799	N (5.54)	2,495	4,596	0.797	N (2.57)	3,118	6,354	0.801	N (1.63)
B	Blood and blood forming organs	655	922	0.902	V (6.17)	1,369	2,096	0.846	D (2.23)	1,661	2,788	0.857	D (1.41)
C	Cardiovascular system	1,537	2,362	0.699	B (5.1)	2,579	4,482	0.702	H (2.11)	3,147	6,185	0.708	N (1.22)
D	Dermatologicals	688	1,007	0.904	J (5.23)	1,498	2,328	0.832	J (3.51)	1,813	3,061	0.851	J (2.02)
G	Genito urinary system and sex hormones	453	571	0.897	H (6.57)	895	1,543	0.898	A (1.69)	1,033	1,923	0.9	
H	Systemic hormonal preparations, excluding sex hormones and insulins	63	70	0.941	V (12.3)	118	152	0.96	B (2.1)	159	212	0.942	
J	Antiinfective for systemic use	652	1,054	0.676	P (8.4)	763	1,071	0.808	D (3.51)	1,112	2,025	0.746	S (1.98)
L	Antineoplastic and immunomodulating agents	806	1,217	0.719	S (5.43)	1,673	3,175	0.393	P (2.66)	1,940	4,066	0.496	P (1.66)
M	Musculo-skeletal system	332	430	0.928	N (5.39)	721	1,117	0.89	N (2.33)	873	1,425	0.906	N (1.7)
N	Nervous system	1,665	2,209	0.66	A (5.54)	2,879	4,864	0.657	A (2.57)	3,450	6,401	0.664	M (1.7)
P	Antiparasitic products, insecticides, and repellents	58	68	0.875	J (8.4)	176	219	0.904	V (3.83)	205	263	0.896	V (2.58)
R	Respiratory system	421	583	0.929	N (4.38)	807	1,239	0.887	C (1.76)	987	1,698	0.896	A (1.18)
S	Sensory organs	875	1,463	0.895	L (5.43)	1,677	2,738	0.833	J (3.34)	1,966	3,812	0.86	P (1.99)
V	Various	133	146	0.972	H (12.3)	358	482	1	P (3.83)	434	586	0.993	P (2.57)
No ATC	No ATC	349	408	0.95		800	1,140	0.942		990	1,463	0.944	

a The ATC-ATC, pair with the highest odds ratio and adjusted *p*-value <0.05.

#### 3.2.5 Identifying ATC category pairs with the highest likelihood of DDIs in static networks

The J-D (Antiinfectives for systemic use—Dermatologicals), A-N (Alimentary tract and metabolism—Nervous system), and M-N (Musculo-skeletal system—Nervous system) pairs were significant and had the highest odds ratios in all three networks (Table 3). Supplementary Table S2 presents all significant ATC-ATC pairs in the static PK, PD, and complete DDI networks.

### 3.3 Evolving network analysis

#### 3.3.1 Evolving power law exponent and assortativity indicate stable scale-free structure, ATC category-based assortativity, and degree-based dissortativity over time

Figure 2 depicts the properties of network evolution. The power law exponent (γ) of the PK DDI network remained stable between 2 and 3, while in the PD and complete DDI networks, *γ* fluctuated until 2001 but has stabilized between 2 and 3 since then (Figure 2A). The ATC category-based assortativities increased over time, suggesting a growing likelihood of DDIs among drugs within the same category (Figure 2B). The degree assortativities declined over time, indicating an increase in the dissortativity of the networks (Figure 2C).

#### 3.3.2 Evolving network clustering coefficients indicate an increasing prevalence of real DDIs among neighbors

The average clustering coefficient gradually increased (within a range of [0,1]) in the PK and complete DDI networks, while it slightly decreased over time in the PD network. However, the clustering coefficients of the PD network were consistently higher than those in the PK and complete networks (Figure 2D). When simulating 100 times with random networks containing the same number of nodes and edges for each year, the average clustering coefficients were not only low but also declined as networks expanded. This finding suggests that DDI networks differ from random networks and evolve towards an increased likelihood of DDIs between neighboring drugs (Supplementary Figure S3).

#### 3.3.3 Evolving network diameter narrows the gap to six degrees of separation

Despite the growth in network size over time (Figures 2E, F), the diameters, which represent the longest length of the shortest paths between any two drugs, have experienced a slight decrease, moving from a range of 8–9 to 7–8 (Figure 2G).

#### 3.3.4 Evolving node and edge counts indicate densification power law in DDI networks

We observed growth in the size of the three networks (in terms of node and edge counts) over the years (Figures 2E, F). The PK, PD, and complete DDI networks exhibited a densification power law with high densification exponents (1.84, 1.75, and 1.9, respectively), signifying that these networks become increasingly dense as they expand in size, thereby raising the likelihood of actual DDIs between two drugs (Figure 3).

**FIGURE 4:**
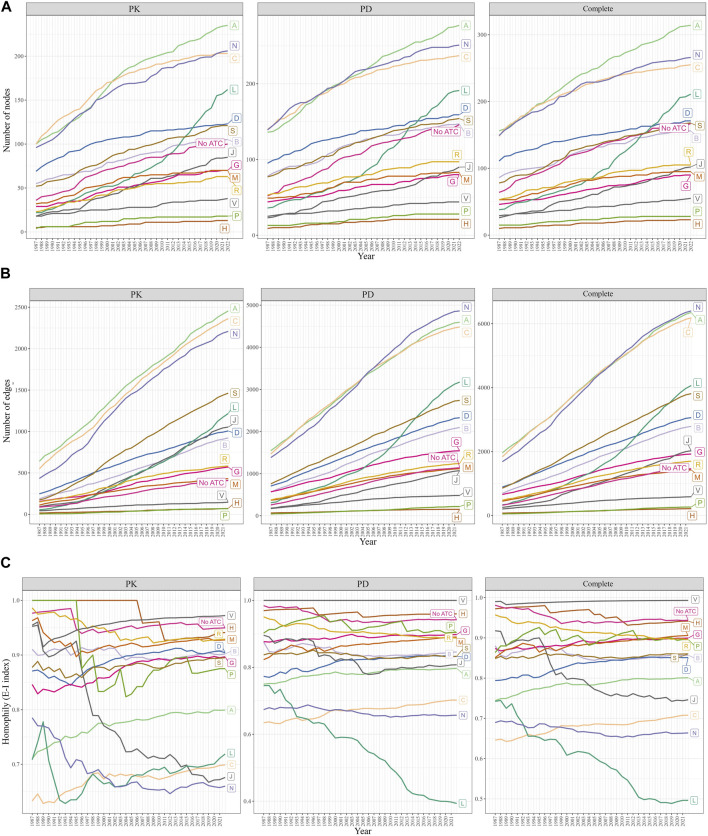
Changes in the size and homophily of the ATC first-level categories. **(A)** Change in the number of nodes. **(B)** Changes in the number of edges. **(C)** Change in the EI-index was measured from 1987 to 2022. A drug may have multiple ATC first-level categories, or none (“No ATC”). The A, C, and N categories were the largest, while the L category grew exponentially since 2007. E-I index scores less than 1 indicated intra-category DDIs, with the L category’s E-I decreasing and the C category’s E-I increasing rapidly.

We observed that the A, C (Cardiovascular system), and N categories consistently had the largest number of nodes (drugs) and edges (DDIs) from 1987 to 2022 (Figures 4A, B). In contrast, the P (Antiparasitic products, insecticides, and repellents) and H (Systemic hormonal preparations, excluding sex hormones and insulins) categories exhibited the smallest number of nodes and edges during the same period. Notably, the number of drugs and DDIs within the L (Antineoplastic and immunomodulating agents) category experienced exponential growth since 2007, while the size of all other ATC categories remained stable, exhibiting a steady growth rate over the years.

#### 3.3.5 Trends in ATC drug category E-I index scores and their implications

The E-I index scores remained below 1 throughout the years (Figure 4C). A significant decrease in the E-I index of the L category was observed in the PD and complete DDI networks, suggesting an increased focus on investigating DDIs within the L category, rather than those involving drugs from L and other categories. The E-I index score for the J category showed a marked downward trend in the PK network. In all three networks, the E-I index scores for the C category experienced the highest growth rate over the years, indicating that the number of DDIs between drugs from the C category and other categories has been increasing more rapidly than the number of DDIs between drugs within the C category itself.

#### 3.3.6 Evolving trends in the ATC category pairs with the highest odds ratios

**FIGURE 5:**
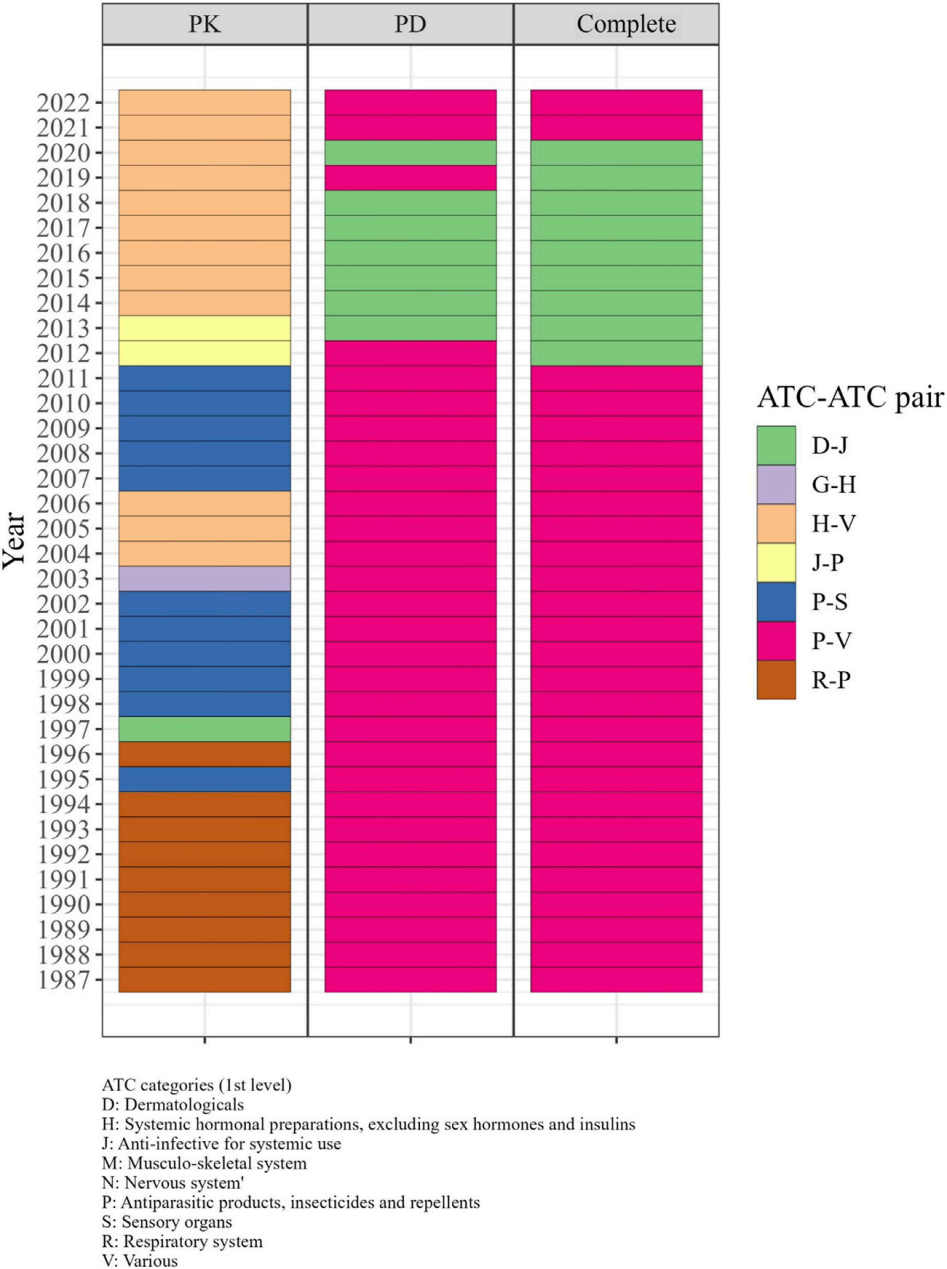
The significant ATC-ATC pairs with the highest odds ratios in each year from 1987 to 2022. In the 1980s–2000s, R-P and P-S pairs in PK, and P-V and D-J pairs in PD and complete networks had the highest odds ratios for DDIs. From 2013–2018, D-J pairs in PD and complete networks and H-V pairs in PK showed the highest odds ratios.

From the 1980s to the early 2000s, the R-P (Respiratory system - Antiparasitic products, insecticides, and repellents) and P-S (Antiparasitic products, insecticides, and repellents—Sensory organs) pairs displayed the highest odds ratio in the PK DDI network, indicating a higher likelihood of DDIs between drugs from these categories compared to other categories. During the same period, P-V (Antiparasitic products, insecticides, and repellents—Various) and D-J pairs exhibited the highest odds ratio in the PD and complete networks from 1987 to 2011 (Figure 5). From 2013 to 2018, the D-J pair had the highest odds ratio in the PD and complete networks, while the H-V pair showed the highest odds ratio in the PK network from 2014 to 2022.

### 3.4 Key influential drugs and trends in DDI networks

#### 3.4.1 Rifampin and Morphine: highly influential drugs in static DDI networks

We found that rifampin ranked first in all three centralities in the PK DDI network, while morphine exhibited the highest values in all three centralities in the PD DDI network. In the complete DDI network, rifampin had the highest degree and eigenvector centrality values, while morphine showed the highest betweenness.

#### 3.4.2 Cimetidine, morphine, ethanol, and rifampin: highly influential drugs over time in evolving DDI networks

**FIGURE 6:**
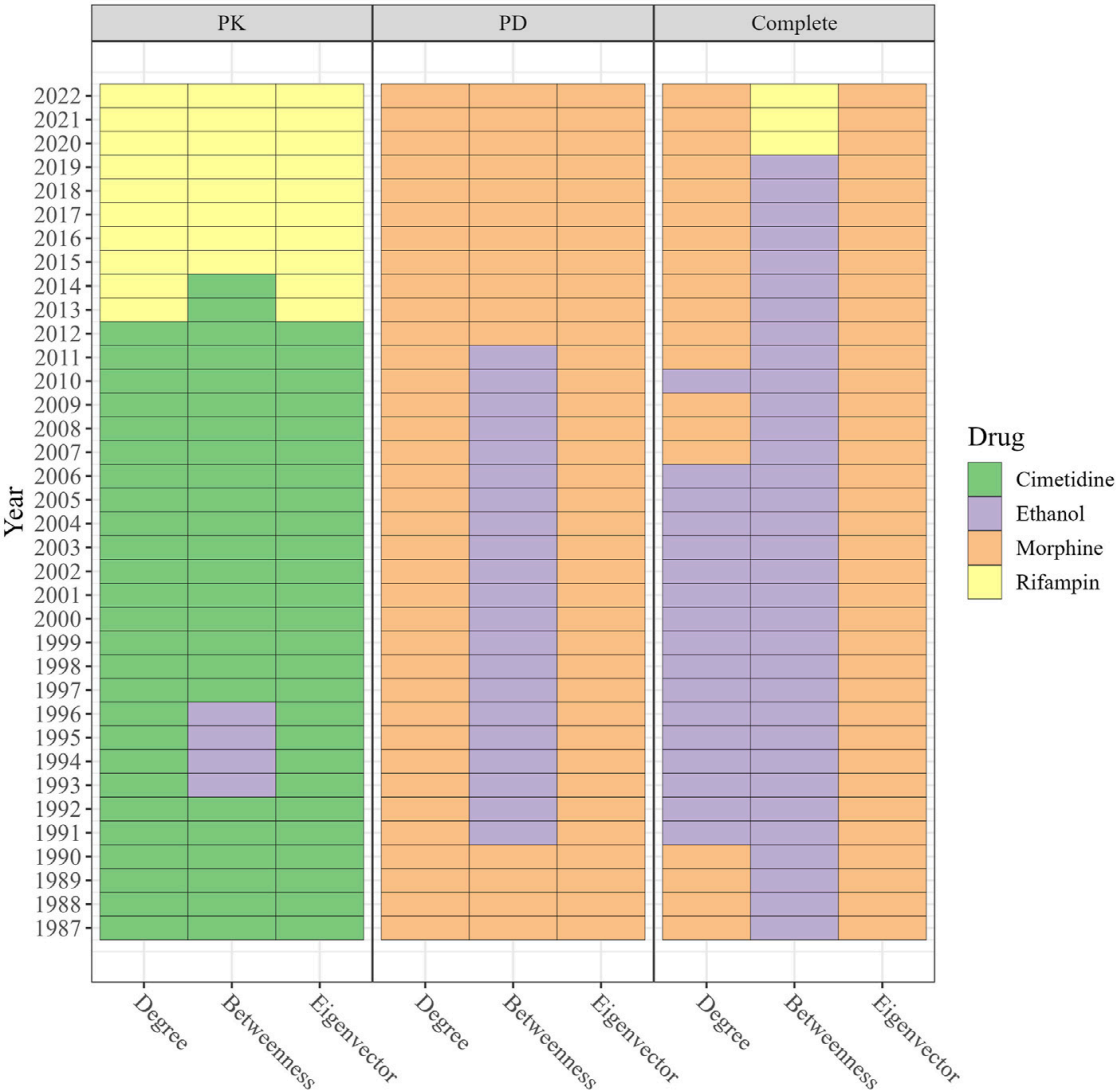
The drugs with the highest degree, betweenness, and eigenvector in PK DDI, PD DDI, and complete DDI networks in each year ranged from 1987 to 2022. The color in the cell represents the drug index. From 1987–2012, cimetidine exhibited the highest degree and eigenvector in the PK network, while rifampin has dominated since 2015. Morphine maintained the highest degree and eigenvectors in the PD and complete networks, with ethanol having the highest betweenness until 2019 when rifampin surpassed it.

Figure 6 displays the drugs with the highest degree, betweenness, and eigenvector centralities for each year. In the PK DDI network from 1987 to 2012, cimetidine demonstrated the highest degree and eigenvector centrality, suggesting that it was extensively investigated for DDIs with numerous other high-degree drugs. Additionally, cimetidine exhibited high betweenness, serving as a connecting point or bridge for various DDIs. Since 2015, rifampin has held the highest degree, betweenness, and eigenvector centrality, indicating its involvement in many DDIs and its interactions with drugs that also have multiple DDIs. By contrast, morphine maintained the highest degree and eigenvector centrality in the PD and complete DDI networks from 1987 to 2022. Ethanol exhibited the highest betweenness in the complete DDI network from 1987 to 2019, while in the most recent 3 years, rifampin emerged with the highest betweenness in the complete DDI network.

#### 3.4.3 Rifampin and fluoxetine: emerging drugs in evolving DDI networks

In the PK and complete DDI network, rifampin exhibited the highest growth rate across degree and betweenness centrality measures. Meanwhile, fluoxetine showed the highest growth rate in eigenvector centrality within the PD and complete DDI networks.

#### 3.4.4 Declining attention on drugs in evolving networks

The eigenvector centrality of cimetidine experienced the greatest decrease in the PK network, while reserpine exhibited the least increase in eigenvector centrality in the PD and complete networks (Table 4). Supplementary Table S3 presents the rate of increase (or decrease) for each drug’s centrality scores in the PK, PD, and complete DDI networks.

**TABLE 4:** The drugs with the highest average, as well as growth rate, in the three types of centrality (degree, betweenness, and eigenvector) over the last 5 years.

		PK	PD	Complete
Largest increase	Degree	Rifampin	Cisplatin	Rifampin
	Betweenness	Rifampin	Morphine	Rifampin
	Eigenvector	Ketoconazole	Fluoxetine	Fluoxetine
Lowest increase (or largest decrease)	Degree	Chlordiazepoxide	Trimethaphan	Meprobamate
	Betweenness	Hexobarbital	Oxygen	Rimantadine
	Eigenvector	Cimetidine	Reserpine	Reserpine

## 4 Discussion

In this study, we employ NLP techniques to extract PK and pharmacodynamic PD DDI evidence from PubMed articles, subsequently characterizing key trends and patterns through static and evolving network analyses. Our findings highlight the scale-free nature of PK and PD networks, with a small number of drugs serving as central hubs, engaging in numerous interactions with other drugs. This observation suggests that the research has focused on specific drugs and their interactions, which could guide future studies to either further explore these central hubs or investigate less-studied drugs. We demonstrate that these networks conform to a densification power law, indicating an exponential growth of DDIs as new drugs are introduced, and emphasizing the increasing complexity of the DDI landscape. Notably, our analysis reveals that drugs within PK and PD networks predominantly belong to the same ATC categories, with fewer interactions observed between drugs from different categories. This insight suggests that DDIs between drugs from distinct ATC categories might be under-explored in the existing literature, warranting further investigation. Moreover, we identify highly influential drugs within static and evolving DDI networks, providing valuable information for future DDI research, clinical practice, and potential areas of focus in drug development.

Our network analysis identified that drugs like rifampin and morphine had high centrality measures, indicating their prominence in DDI research. Rifampin is an antibiotic agent used for treating tuberculosis and other bacterial infections. It is frequently administered in conjunction with other antituberculosis drugs or other families of drugs and has a significant potential for drug interactions due to its well-known induction of drug metabolism through cytochrome P450 (CYP)1A2, CYP2C8, CYP2C9, CYP2C19, CYP3A4, and some glucuronidation pathways (Venkatesan, 1992). It is difficult to predict which medications will be affected by the selective enzyme-induction effect of rifampin (Venkatesan, 1992). Morphine, on the other hand, is the first-choice opioid for the management of cancer pain according to the World Health Organization (WHO) guidelines (Staff and Organization, 1996). The risk of DDIs is high in cancer patients due to a large number of concomitant drugs (Kotlinska-Lemieszek et al., 2014). In the static PD and complete networks, the most researched DDI was morphine–naloxone. This DDI was intensively studied between the 1980s and the early 2000s. Morphine is the classic opioid agonist that provides considerable analgesia and respiratory depression (Sartain et al., 2003), while naloxone is an opioid antagonist capable of reversing the powerful opioid effects of morphine and inducing the opposite effect of hyperalgesia and reversing respiratory depression (Westbrook and Greeley, 1990). Morphine and naloxone were the most notable opioid drugs studied in the past for pain modulation, opioid tolerance, and opioid dependency, especially since naloxone is considered an antidote for morphine and other opioids (Westbrook and Greeley, 1990).

Our investigation also indicated dynamic changes in the DDI research over time using an evolving network analysis, which the traditional static network analysis is unable to provide. While the PK, PD, and complete DDI networks were scale-free, they also followed the densification power law, wherein the number of DDIs grows faster than the number of drugs—networks become denser over time. Despite the network size growth over time, the average local clustering coefficient of all three networks remains high, indicating that the DDI networks are developing small-world network characteristics. This suggests that the drugs studied in DDI research trends are becoming increasingly interconnected and that the scientific community is becoming more adept at examining and comprehending the complex relationships between drugs. Degree assortativity has decreased over the years, while ATC-group assortativity has increased, suggesting that the number of connections between drugs with a high and low degree has been increasing (decreased degree assortativity), and the number of connections between drugs in the same ATC categories has been increasing (increased ATC assortativity).

Even though the A, C, and N categories still comprised the majority of DDI research, the ATC-level analysis revealed that the number of drugs and DDIs in the L category has increased dramatically since 2002. This may be because combination therapy, a treatment modality that combines two or more therapeutic agents, is a cornerstone of cancer therapy (Bayat Mokhtari et al., 2017). This also explains the decreasing trend in the E-I index of the L category, which indicates that the DDIs between drugs in the same L category have recently been investigated. It is worth noting that the number of DDI studies may be influenced by prescription frequency. For example, According to Bodenreider and Rodriguez (2014), despite the fact that the dataset was based on emergency room patients for 3 months in 2011, the A, C, and N categories were the most commonly prescribed drug categories, so the sheer number of DDIs found in categories A, C, and N might be inflated due to the fact that these drugs are more commonly prescribed, leading to more observations and subsequent publications. However, we have found that high-frequency prescribed drugs are not always investigated in DDI research, and low-frequency prescribed drugs can also be highly investigated for DDIs. For instance, the L category drugs were prescribed at a very low rate, but our research showed that the number of L category related DDI studies was very high in 2011. Conversely, the H category drugs were frequently prescribed, but their DDIs were rarely investigated in 2011. These findings suggest that other factors, such as safety concerns or emerging research interests, may play a role in driving DDI research beyond drug prescription frequency alone.

Between 1987 to 2012, cimetidine had the highest degree, betweenness, and eigenvector centralities in the PK DDI network, but it was replaced by rifampin. Cimetidine has numerous drug interactions due to its nonselective inhibition of cytochrome P450 enzymes (Levine and Bellward, 1995). The introduction of longer-acting H2 receptor antagonists with fewer side effects and drug interactions has diminished the usage of cimetidine, and it is no longer one of the most regularly used H2 receptor antagonists. Similarly, the therapeutic applications of trimethaphan (a vasodilator), which showed the lowest eigenvector increase in the PK network, are extremely limited due to competition from newer drugs with more selective actions and effects produced (Wilkins et al., 2007).

Rimantadine demonstrated the greatest decrease in betweenness in the complete network, which may be due to the fact it is not recommended for use in the United States since 2009 because of widespread antiviral resistance to this class of antivirals among circulating flu A viruses (Bloom et al., 2010). Colistin–meropenem was the most actively researched DDI in the PD and complete DDI networks over the past 5 years. Numerous studies demonstrated that the combination of different antibiotics with colistin, such as meropenem produced favorable results (Biancofiore et al., 2007). Recently, researchers have questioned whether the colistin–meropenem combination has a synergistic effect (better than monotherapy) against bacteria (Soudeiha et al., 2017). The controversial opinions expressed by researchers may have prompted the recent active investigation of this DDI.

Despite its contributions, our study has several limitations. First, although we employed the ScispaCy and SciFive models, the results may include false positives and overlook articles due to false negatives, as the model is not perfect. While we confirmed the performance of the NLP models through manual evaluations of a set of randomly selected DDIs, a thorough manual examination of all DDI sentences would be necessary to improve the quality of the results. Second, some relevant publications may have been excluded from this study if they did not fall within the search criteria. For instance, our conclusions are based on the assumption that all DDI articles contained at least one sentence with at least two drug entities in their abstracts. However, there may be DDI articles that lack such a sentence or contain a sentence with at least two drug entities only in the full text, and our study would not include these articles. However, extracting DDIs from full-text articles with acceptable performance is challenging for NLP models. Despite the existence of advanced NLP models such as SciFive, knowledge graphs, and large language models, their performance in extracting DDIs from full-text articles is unknown. Furthermore, many sentences in full-text articles describe or introduce DDIs from cited papers, which can skew the results. Third, we acknowledge that the 5-year investigation window size we chose to inform readers about recent DDI research trends was arbitrary. Even though we believed that a 5-year period could provide recent trends in DDI research because longer timeframes could capture more historical trends and shorter timeframes could not reveal trends adequately, the selection of a 5-year investigation window size may not fully represent the recent DDI research trend. As a result, the recent trends in this paper should be interpreted using the 5-year investigation window. Lastly, the quality of DDI evidence extracted from the literature is dependent on the quality of the original research, which may be limited or inconsistent. This may lead to variability in the quality and relevance of DDI evidence extracted from the literature, potentially resulting in incomplete or biased analyses. Future studies should focus on enhancing data quality, including the manual curation of DDI evidence from published literature, to develop high quality DDI networks. Incorporating the clinical implications of DDIs into network analysis is also crucial. This would highlight the clinical significance of these interactions, providing insights that could be instrumental in optimizing patient care.

## Data Availability

The datasets presented in this study can be found in online repositories. The names of the repository/repositories and accession number(s) can be found in the article/Supplementary Material.

## Abbreviations

A, Alimentary tract and metabolism; ATC, Anatomical Therapeutic Chemical; B, Blood and blood forming organs; C, Cardiovascular system; CYP, Cytochrome P450; D, Dermatologicals; DDI, Drug-Drug Interaction; DPL, Densification Power Law; G, Genito urinary system and sex hormones; H, Systemic hormonal preparations, excluding sex hormones and insulins; J, Antiinfective for systemic use; L, Antineoplastic and immunomodulating agents; M, Musculo-skeletal system; N, Nervous system; NA, Network Analysis; NER, Name Entity Recognition; NLP, Natural Language Processing; P, Antiparasitic products, insecticides, and repellents; PD, Pharmacodynamic; PK, Pharmacokinetic; R, Respiratory system; RE, Relation Extraction; S, Sensory organs; SOTA, State-Of-The-Art; T5, Text-To-Text Transfer Transformer; UMLS, Unified Medical Language System; V, Various; WHO, World Health Organization.

## References

[B1] Bayat Mokhtari R. Homayouni T. S. Baluch N. Morgatskaya E. Kumar S. Das B. (2017). Combination therapy in combating cancer. Oncotarget 8, 38022–38043. 10.18632/oncotarget.16723 28410237 PMC5514969

[B2] Becker M. L. Kallewaard M. Caspers P. W. Visser L. E. Leufkens H. G. Stricker B. H. (2007). Hospitalisations and emergency department visits due to drug-drug interactions: a literature review. Pharmacoepidemiol Drug Saf. 16, 641–651. 10.1002/pds.1351 17154346

[B3] Biancofiore G. Tascini C. Bisa M. Gemignani G. Bindi M. L. Leonildi A. (2007). Colistin, meropenem and rifampin in a combination therapy for multi-drug-resistant Acinetobacter baumannii multifocal infection. A case report. Minerva Anestesiol. 73, 181–185. 17159765

[B4] Bloom J. D. Gong L. I. Baltimore D. (2010). Permissive secondary mutations enable the evolution of influenza oseltamivir resistance. Science 328, 1272–1275. 10.1126/science.1187816 20522774 PMC2913718

[B5] Bodenreider O. Rodriguez L. M. (2014). Analyzing U.S. Prescription lists with RxNorm and the ATC/DDD index. AMIA Annu. Symp. Proc. 2014, 297–306. 25954332 PMC4419961

[B6] Bodenreider O. (2004). The unified Medical Language System (UMLS): integrating biomedical terminology. Nucleic Acids Res. 32, D267–D270. 10.1093/nar/gkh061 14681409 PMC308795

[B7] Boyce R. Gardner G. Harkema H. (2012). “Using natural language processing to identify pharmacokinetic drug-drug interactions described in drug package inserts,” in Proceedings of the 2012 Workshop on Biomedical Natural Language Processing, Montreal, Quebec, Canada, 2012 May 07 (Association for Computational Linguistics), 206–213.

[B8] Chen Y. Yan C. Patel M. B. (2020). Network analysis subtleties in ICU structures and outcomes. Am. J. Respir. Crit. Care Med. 202, 1606–1607. 10.1164/rccm.202008-3114LE 32931298 PMC7706157

[B9] Csardi G. Nepusz T. (2006). The igraph software package for complex network research. InterJournal, complex Syst. 1695, 1–9.

[B10] Duda S. Aliferis C. Miller R. Statnikov A. Johnson K. (2005). Extracting drug-drug interaction articles from MEDLINE to improve the content of drug databases. AMIA Annu. Symp. Proc. 2005, 216–220. 16779033 PMC1560879

[B11] Honnibal M. Montani I. (2017). spaCy 2: Natural language understanding with Bloom embeddings, convolutional neural networks and incremental parsing. appear 7, 411–420.

[B12] Jeong E. Ko K. Oh S. Han H. W. (2017). Network-based analysis of diagnosis progression patterns using claims data. Sci. Rep. 7, 15561. 10.1038/s41598-017-15647-4 29138438 PMC5686166

[B13] Kleinfeld J. (2002). Could it be a big world after all? The six degrees of separation myth. Society 12, 5–2.

[B14] Kotlinska-Lemieszek A. Paulsen O. Kaasa S. Klepstad P. (2014). Polypharmacy in patients with advanced cancer and pain: a European cross-sectional study of 2282 patients. J. Pain Symptom Manage 48, 1145–1159. 10.1016/j.jpainsymman.2014.03.008 24780183

[B15] Krackhardt D. Stern R. N. (1988). Informal networks and organizational crises: An experimental simulation. Soc. Psychol. Q. 51, 123–140. 10.2307/2786835

[B16] Leskovec J. Kleinberg J. Faloutsos C. (2007). Graph evolution: Densification and shrinking diameters. ACM Trans. Knowl. Discov. Data (TKDD) 1, 2–es. 10.1145/1217299.1217301

[B17] Leskovec J. Kleinberg J. Faloutsos C. (2005). “Graphs over time: densification laws, shrinking diameters and possible explanations,” in Proceedings of the eleventh ACM SIGKDD international conference on Knowledge discovery in data mining, 177–187.

[B18] Levine M. Bellward G. D. (1995). Effect of cimetidine on hepatic cytochrome P450: evidence for formation of a metabolite-intermediate complex. Drug Metab. Dispos. 23, 1407–1411. 8689952

[B19] Nebert D. W. Russell D. W. (2002). Clinical importance of the cytochromes P450. Lancet 360, 1155–1162. 10.1016/S0140-6736(02)11203-7 12387968

[B20] Neumann M. King D. Beltagy I. Ammar W. (2019). ScispaCy: Fast and robust models for biomedical natural language processing. *arXiv preprint arXiv:1902.07669* .

[B21] Nigam S. K. (2015). What do drug transporters really do? Nat. Rev. Drug Discov. 14, 29–44. 10.1038/nrd4461 25475361 PMC4750486

[B22] Niu J. Straubinger R. M. Mager D. E. (2019). Pharmacodynamic drug-drug interactions. Clin. Pharmacol. Ther. 105, 1395–1406. 10.1002/cpt.1434 30912119 PMC6529235

[B23] Phan L. N. Anibal J. T. Tran H. Chanana S. Bahadroglu E. Peltekian A. (2021). Scifive: A text-to-text transformer model for biomedical literature. *arXiv preprint arXiv:2106.03598* .

[B24] Qu Y. Guan X. Zheng Q. Liu T. Zhou J. Li J. (2015). Calling network: A new method for modeling software runtime behaviors. ACM SIGSOFT Softw. Eng. Notes 40, 1–7. 10.1016/j.pupt.2015.07.004

[B25] Qu Y. Zheng Q. Liu T. Li J. Guan X. (2014). In-depth measurement and analysis on densification power law of software execution. Proc. 5th Int. Workshop Emerg. Trends Softw. Metrics, 55–58. 10.1145/2593868.2593878

[B26] Raffel C. Shazeer N. Roberts A. Lee K. Narang S. Matena M. (2020). Exploring the limits of transfer learning with a unified text-to-text transformer. J. Mach. Learn. Res. 21, 1–67.34305477

[B27] Sartain J. B. Barry J. J. Richardson C. A. Branagan H. C. (2003). Effect of combining naloxone and morphine for intravenous patient-controlled analgesia. Anesthesiology 99, 148–151. 10.1097/00000542-200307000-00024 12826854

[B28] Segura-Bedmar I. MartïNEZ FernãNDEZ P. Herrero Zazo M. (2013). Semeval-2013 task 9: Extraction of drug-drug interactions from biomedical texts (ddiextraction 2013). Association for Computational Linguistics.

[B29] Soudeiha M. A. H. Dahdouh E. A. Azar E. Sarkis D. K. Daoud Z. (2017). *In vitro* evaluation of the colistin-carbapenem combination in clinical isolates of A. Baumannii using the checkerboard, etest, and time-kill curve techniques. Front. Cell Infect. Microbiol. 7, 209. 10.3389/fcimb.2017.00209 28596943 PMC5442352

[B30] Staff W. H. O. Organization W. H. (1996). Cancer pain relief: With a guide to opioid availability. World Health Organization.

[B31] Van Mil J. W. (2016). Stockley's drug interactions 11th edition. Int. J. Clin. Pharm. 38, 1003–1004. 10.1007/s11096-016-0325-2 27241340

[B32] Venkatesan K. (1992). Pharmacokinetic drug interactions with rifampicin. Clin. Pharmacokinet. 22, 47–65. 10.2165/00003088-199222010-00005 1559307

[B33] Westbrook R. Greeley J. (1990). Some effects of the opioid antagonist, naloxone, upon the rat's reactions to a heat stressor. Q. J. Exp. Psychol. Sect. B 42, 1–40. 10.1080/14640749008401869 2158122

[B34] Wilkins B. W. Hesse C. Sviggum H. P. Nicholson W. T. Moyer T. P. Joyner M. J. (2007). Alternative to ganglionic blockade with anticholinergic and alpha-2 receptor agents. Clin. Auton. Res. 17, 77–84. 10.1007/s10286-006-0387-7 17160588

[B35] Yan C. Zhang X. Gao C. Wilfong E. Casey J. France D. (2021). Collaboration structures in COVID-19 critical care: Retrospective network analysis study. JMIR Hum. Factors 8, e25724. 10.2196/25724 33621187 PMC7942392

