# Supplementary material for: Revealing the dynamic landscape of drug-drug interactions through network analysis
Source: Front Pharmacol. 2023 Oct 3;14:1211491. doi: 10.3389/fphar.2023.1211491 (PMC10583566; doi:10.3389/fphar.2023.1211491)
Supplement: Supplementary file 4 [file DataSheet1.docx]

**Revealing the Dynamic Landscape of Drug-Drug Interactions through Network Analysis**

**Eugene Jeong, MS^1^, Bradley Malin, PhD^1,2,3^, Scott D Nelson, PharmD, MS^1^, Yu Su, PhD^4^, Lang Li, PhD^5^, You Chen, PhD^1,3*^**

^1^Department of Biomedical Informatics, School of Medicine, Vanderbilt University Medical Center, Nashville, Tennessee, United States

^2^Department of Biostatistics, School of Medicine, Vanderbilt University Medical Center, Nashville, Tennessee, United States

^3^Department of Computer Science, School of Engineering, Vanderbilt University, Nashville, Tennessee, United States

^4^Department of Computer Science and Engineering, College of Engineering, The Ohio State University, Columbus, Ohio, United States

^5^Department of Biomedical Informatics, College of Medicine, The Ohio State University, Columbus, Ohio, United States

*** Correspondence:**

You Chen, Ph.D.

you.chen@vanderbilt.edu

# Supplementary Figures

**
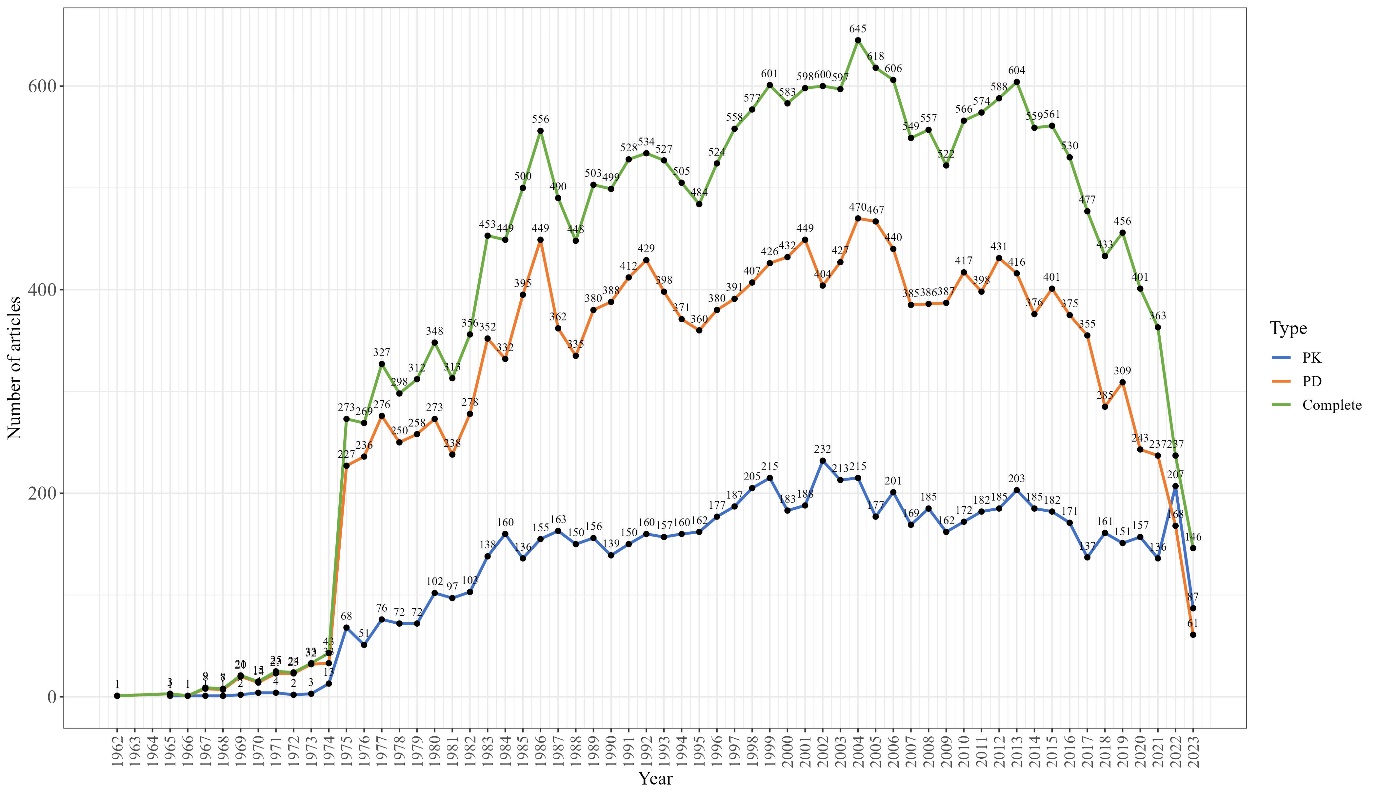
**

**Supplementary Figure 1.** The number of PK, PD, and All DDI articles published in each year between 1962 and 2023. The blue line represents the number of PK DDI articles, the orange line represents the number of PD DDI articles, and the green line represents the number of complete DDI articles.


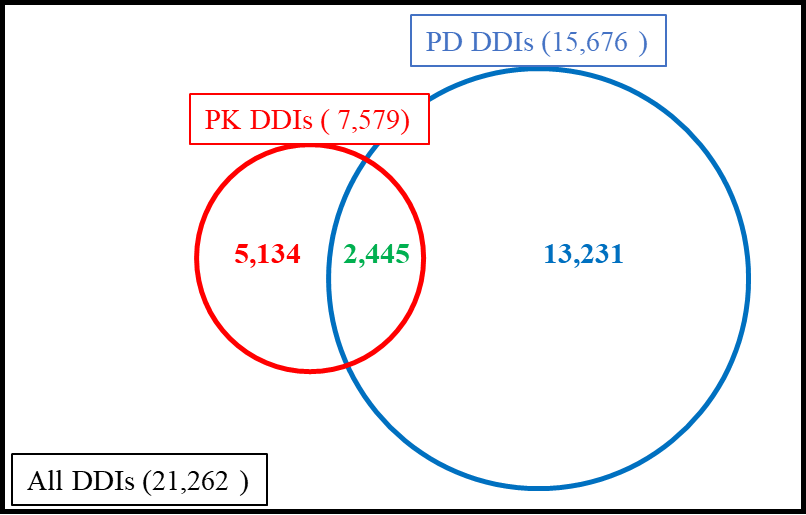


Supplementary Figure 2. DDI overlap between PK DDIs and PD DDIs.

*
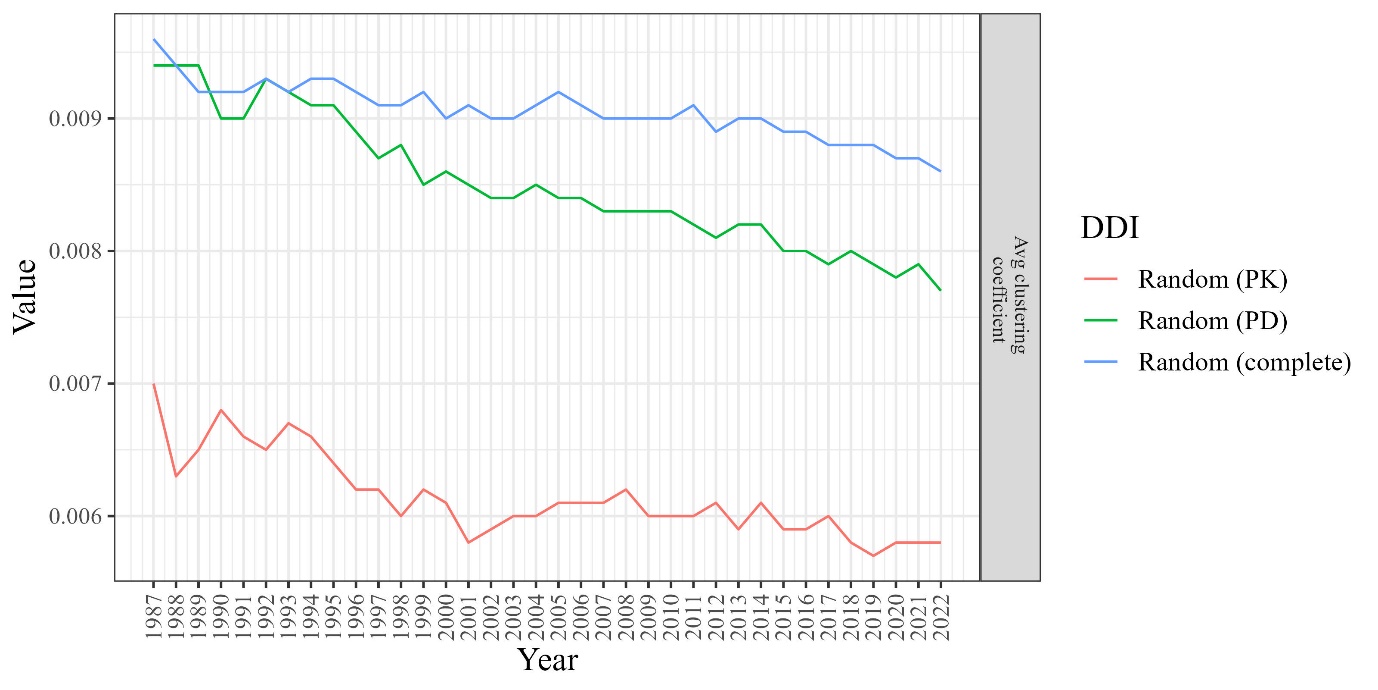
*

Supplementary Figure 3. Changes in the average local clustering coefficient of the random DDI networks. The average clustering coefficients decreased as networks expanded.
